# The journey of nisin development in China, a natural-green food preservative

**DOI:** 10.1007/s13238-015-0214-9

**Published:** 2015-09-30

**Authors:** Jie Zhang, Jin Zhong

**Affiliations:** Institute of Microbiology, Chinese Academy of Sciences, Beijing, 100101 China

In 2005, the project “Research and Development of Nisin” led by Prof. Liandong Huan was awarded the National Science & Technology Progress Prize (Second Class Prize) and China National Food Industry Association (CNFIA) Science & Technology Prize (Special Prize).

Nisin is a lantibiotic (bacteriocin containing unusual amino acids) (Fig. 1) mainly produced by gram-positive lactic acid bacteria, such as *Lactococcus lactis*. It shows a broad spectrum of antimicrobial activity against many food-borne pathogens such as *Listeria monocytogenes*, *Staphylococcus aureus*, and *Clostridium botulinum*, at very low levels (nmol/L range). In addition, nisin is heat-stable, non-toxic, and sensitive to digestive proteases. So far, nisin is the only bacteriocin approved by both United States FDA and the WHO for use as a food preservative and it is utilized in more than 50 countries around the world. It is widely used in canned foods, dairy products, meat products, alcoholic beverages, etc.

**Figure 1:**
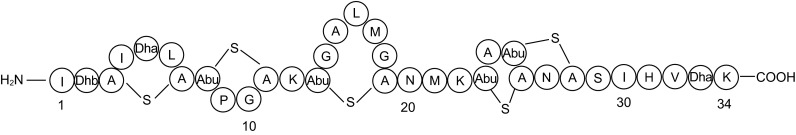
The primary structure of nisin Z

Nisin was first discovered in milk in 1928, around the same time as the first antibiotic penicillin was discovered. In the 1950s, nisin was commercially produced by Aplin & Barrett in Britain. The study of nisin in China began in the late 1980s. At the end of 1987, scientists from the Institute of Microbiology, Chinese Academy of Sciences (IMCAS), realized that nisin was a product of great potential for food safety and human health. However, the production of nisin exclusively belonged to only one foreign company at that time. The former director of IMCAS, Prof. Yugu Xue (Fig. 2), showed great foresight and immediately initiated a research project on nisin. Under her leadership, researchers from the Department of Genetics pioneered fundamental research on nisin in China. Based on genetic analysis, a new method to screen the producing strain of nisin was developed (Huan et al., 1997), and an over-producer strain was obtained by physical and chemical mutagenesis and several rounds of mutation breeding. Moreover, the group also cloned the biosynthetic gene cluster of nisin and found that the product was a new type (nisin Z), which is different from the foreign nisin A.

**Figure 2:**
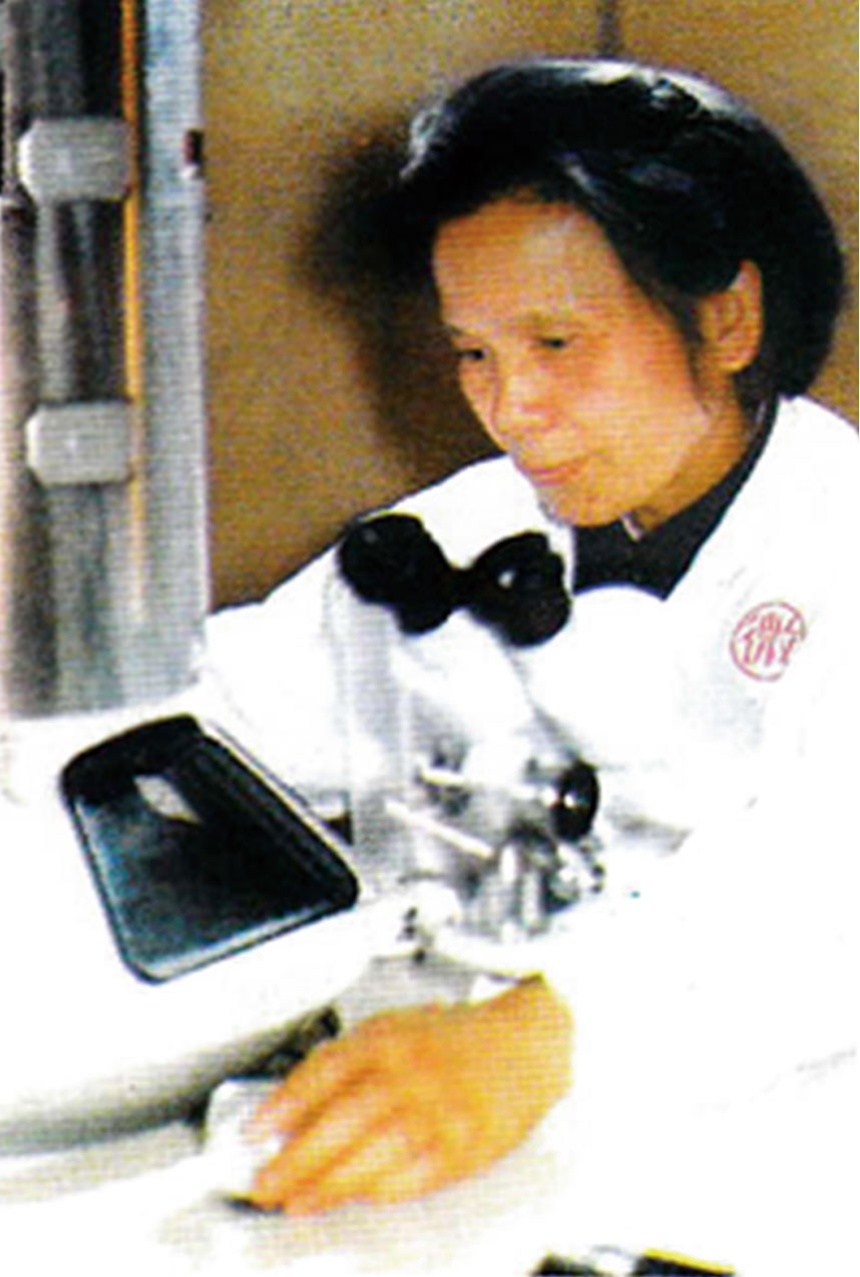
Prof. Yugu Xue (1982)

During development, the group led by Prof. Liandong Huan (Fig. 3) innovatively used cheap plant peptone and yeast powder as fermentation medium, which was much cheaper than the milk used by the foreign company. The lab trials indicated that they had successfully produced nisin. Large scale production pilot trials were carried out in Zhejiang Tiantai Silver-Elephant Bio-chemical Engineering factory (the former Zhejiang Silver-Elephant bioengineering Co., Ltd.) and succeeded two years later with much effort of the researchers. In 1995, the first production line of nisin with the ability to produce 20 tons every year was built in China. In 1996, the project “Special Preservative Nisin” was included in National Science and Technique Foundation during the “9th Five-Year Plan”. During this time, the production of nisin was extended to industrial scale. Furthermore, with the support of National Science and Technique Foundation during the “10th Five-Year Plan” and national high-tech industrialization demonstration project, the producing strain of nisin and the techniques for fermentation and extraction were further improved, resulting in a large increase in production and at a lower cost. Consequently, in 2005, the biggest modern factory of the world was founded in Tiantai county of Zhejiang province. Its production of nisin reached to 150 tons each year and the market share was also the highest of the world, creating great benefits for economy and society.

**Figure 3:**
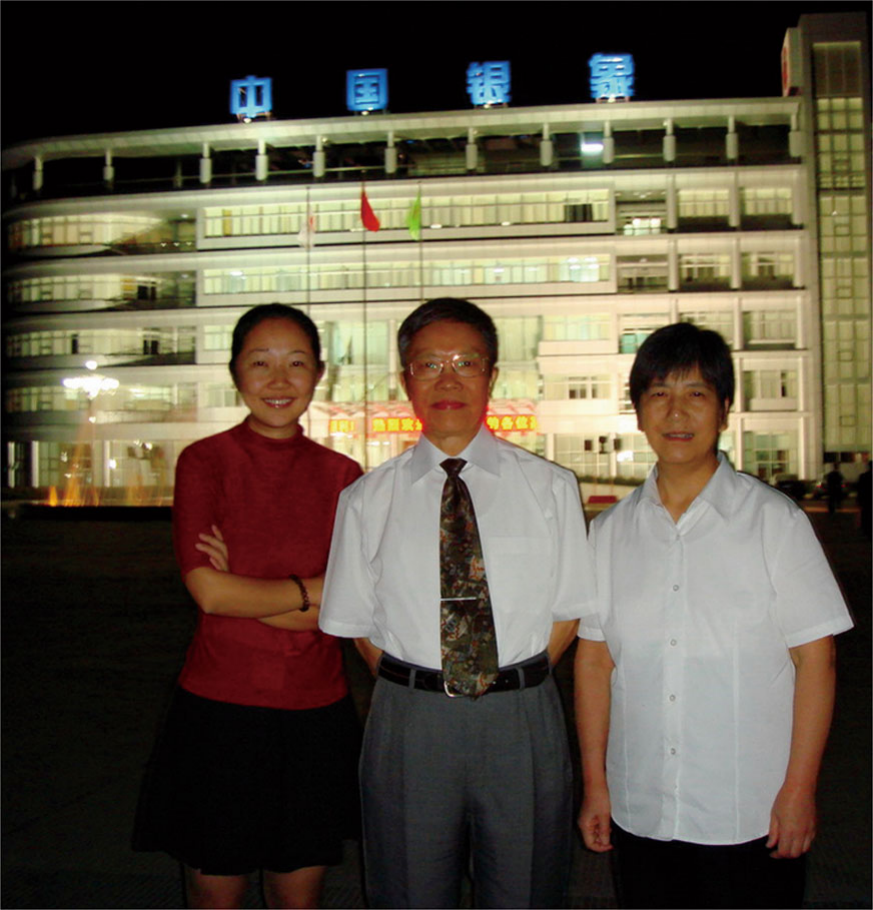
Prof. Liandong Huan (middle), associate Prof. Xiuzhu Chen (right), and Prof. Jin Zhong (left) at Silver-Elephant company

The process of research and development of nisin was tortuous. At first, the product was too advanced and expensive to people, leading to poor sales. The Silver-Elephant company was also in serious trouble with financing. Meanwhile, the institute gave a great deal of trust and support, rejecting the requests to sell the intellectual property from many domestic and foreign companies. Moreover, nisin appeared to have a limited scope of application during the development. However, with the institute inviting experts of other institutions to participate in the application tests of nisin, the application of nisin in low temperature meat products, dairy products, and canned food was accelerated. Eventually, many well-known food companies such as Shuanghui, Wahaha, Sanyuan, and Huiyuan, became fixed clients.

The success of research and development of nisin was attributed to a hard-working team and the fruits of their labour resulted from the effort of several generations of scientists. Prof. Yugu Xue, the pioneering and leading scientist at nearly seventy-years old, continued to make outstanding contributions to the project and Associate Prof. Xiuzhu Chen (Fig. 3) devoted many hours of her free-time to lead the staffs from the Silver-Elephant company to solve production problems. After untold hardships, the fermentation titer was increased from 3000 units to over 8000 units per milliliter. The work reached the leading level at home and abroad and made a great step forward for the research and development of nisin. Besides, many graduate students also contributed to the project. For example, Ying Cai was involved in the establishment of method to directly screen the producing strain of nisin; Haiqing Hu cloned the complete gene cluster of nisin; Jing Yuan constructed nisin mutants with broader antimicrobial spectra (Yuan et al., 2004), and so on. The related work on nisin has been published in more than 30 research papers and obtained 5 authorized national invention patents.

The research and development of nisin is still going on in IMCAS. On the basis of previous work, the group led by Prof. Jin Zhong (Fig. 3) has clarified the structure-activity relationships, the mode of action, and the resistance mechanism of nisin (Sun et al., 2009). They also have worked on the resolution of the biosynthetic machinery, especially the self-induction molecular mechanism (Teng et al., 2014). Additionally, they have exploited many potential lantibiotics from microbial resources or genomic resources. All these works will benefit the wide application of nisin-like active substances in food, medicine, and other fields.

## References

[CR1] Huan LD, Chen XZ, Cai Y, Zhuang ZH, Xue YG (1997) Directional screening of nisin-producing *Lactococcus lactis* and identification of its product. Acta Microbiol Sin 37:292–300. (还连栋, 陈秀珠, 才迎, 庄增辉, 薛禹谷. (1997). 乳链菌肽产生菌的定向筛选及发酵产物的鉴定. 微生物学报, 37, 292–300)

[CR3] Sun Z, Zhong J, Liang XB, Liu JL, Chen XZ, Huan LD (2009). Novel mechanism for nisin resistance via proteolytic degradation of nisin by the nisin resistance protein NSR. Antimicrob Agents Chemother.

[CR4] Teng KL, Zhang J, Zhang X, Ge XX, Gao Y, Wang J, Lin YH, Zhong J (2014). Identification of ligand specificity determinants in lantibiotic bovicin HJ50 and the receptor BovK, a multitransmembrane histidine kinase. J Biol Chem.

[CR2] Yuan J, Zhang ZZ, Chen XZ, Yang W, Huan LD (2004). Site-directed mutagenesis of the hinge region of nisin Z and properties of nisin Z mutants. Appl Microbiol Biotechnol.

